# Cytokines and Chemokines in SARS-CoV-2 Infections—Therapeutic Strategies Targeting Cytokine Storm

**DOI:** 10.3390/biom11010091

**Published:** 2021-01-12

**Authors:** Alexandra Pum, Maria Ennemoser, Tiziana Adage, Andreas J. Kungl

**Affiliations:** 1 Institute Of Pharmaceutical Sciences, Karl-Franzens-University Graz, Schubertstrasse 1, 8010 Graz, Austria; alexandra.pum@uni-graz.at (A.P.); maria.ennemoser@uni-graz.at (M.E.); 2 Brain Implant Therapeutics (BIT) Pharma, Leonhardstrasse 109, 8010 Graz, Austria; tadage@hotmail.com; 3 Antagonis Biotherapeutics GmbH, Strasserhofweg 77, 8045 Graz, Austria

**Keywords:** chemokines, SARS-CoV-2, COVID-19, coronavirus, cytokine storm

## Abstract

The recently identified severe acute respiratory syndrome coronavirus 2 (SARS-CoV-2) virus, the cause of coronavirus disease (COVID-19) and the associated ongoing pandemic, frequently leads to severe respiratory distress syndrome and pneumonia with fatal consequences. Although several factors of this infection and its consequences are not completely clear, the presence and involvement of specific chemokines is undoubtedly crucial for the development and progression of COVID-19. Cytokine storm and the often-resulting cytokine release syndrome (CRS) are pathophysiological hallmarks in COVID-19 infections related to its most severe and fatal cases. In this hyperinflammatory event, chemokines and other cytokines are highly upregulated and are therefore not fulfilling their beneficial function in the host response anymore but causing harmful effects. Here, we present the recent views on the involvement of chemokines and selected cytokines in COVID-19 and the therapeutics currently in clinical development targeting or interfering with them, discussing their potentials in the treatment of COVID-19 infections.

## 1. Introduction

In December of 2019, a novel strain of beta coronavirus named severe acute respiratory syndrome coronavirus 2 (SARS-CoV-2) emerged as the causative agent of coronavirus disease (COVID-19), which rapidly spread throughout the world and was declared a global pandemic by the WHO in March 2020 [1]. Advanced age and the presence of pre-existing comorbidities are considered risk factors for a more severe course and mortality of COVID-19 [2,3]. As of November 2020, the European Centre for Disease Prevention and Control has reported more than 50 million cases of COVID-19, including more than 1.3 million deaths worldwide. Moreover, many infected people remain asymptomatic while being contagious, making it even more challenging to control the rapid spread of SARS-CoV-2 [4].

Besides the asymptomatic courses, SARS-CoV-2 also causes a remarkable variety of clinical symptoms, including fever (43.8%; 88.7% after hospitalization), fatigue, and dry cough (67.8%). Lymphocytopenia (83.2%) and ground-glass opacity in computed chest tomography scans (56.4%) were the most common diagnostic findings in intensive care unit (ICU) patients on admission [5]. Driven by the increasing daily number of confirmed cases and the results of clinical trials, remdesivir (Gilead Sciences, Inc., Foster City, California, USA) was granted an emergency use authorization (EUA) from the US Food and Drug Administration (FDA) as the first treatment of COVID-19 for patients requiring hospitalisation [6]. The FDA has further issued a EUA for the combination therapy of monoclonal antibodies casirivimab and imdevimab (also known as REGN10933 and REGN10987, Regeneron Pharmaceuticals, Tarrytown, NY, USA) for the administration in the treatment of mild to moderate COVID-19 in adults and paediatric patients in November 2020 [6].

COVID-19 physiopathology is not yet completely understood, and the particular underlying reasons for the extremely varying degrees of disease progression need to be further investigated. For the two phylogenetically related SARS-CoV and the Middle East respiratory syndrome (MERS-CoV) diseases, it is known that specific cytokine and chemokine serum levels are increased [7,8]. Chemokines represent a group of chemotactic cytokines secreted from a variety of cell types, following viral or bacterial infectious events and act by binding G-protein coupled seven-transmembrane receptors (GPCRs) on the surface of leukocytes. This axis shows promiscuity because some chemokines can activate more than one receptor and some receptors can be activated by more than one chemokine. In addition to binding to the typical GPCR chemokine receptors, chemokines also bind another class of biomolecules called the glycosaminoglycans (GAGs), which are attached to core proteins of cell-surfaces or extracellular matrix proteoglycans. GAGs present and activate chemokines to make them fit for the purpose of mobilizing and recruiting various immune cells [9,10]. The acute respiratory distress syndrome (ARDS), which often occurs in the more severe pathological manifestations of COVID-19, is characterised by the increased presence of infiltrated leukocytes. This immune cell migration can result in severe respiratory failure with consequent sepsis, trauma, or other clinical events [11].

While the production and release of cytokines in healthy individuals underlie an essential balance of inflammatory and homeostatic factors, in the case of inflammatory diseases, uncontrolled overproduction of cytokines can often occur, having fatal consequences for the organisms. Therefore, the interest in investigating the role of dysregulated chemokines in SARS-CoV-2 infection has emerged. Currently, a very often occurring and much-discussed term in this context is the so-called cytokine storm phenomenon, appearing as an event characterised by fulminant hyperactivity of immune cells producing pro-inflammatory cytokines, referred to hypercytokinemia, which can lead to severe or even life-threatening cases of multiple organ failures [12]. 

From a molecular point of view, SARS-CoV-2 enters target cells by binding of viral spike (S) surface protein to angiotensin-converting enzyme 2 (ACE2) receptors of respiratory epithelial cells [13]. GAGs, more precisely heparan sulphate, act as co-receptor for viral entry [14]. This rapidly induces expression of Th1 cells, which represents a fast, innate immune response and further activates proinflammatory mediators such as interleukin-6 (IL-6), granulocyte macrophage colony-stimulating factor, and CD14+CD16+ monocytes. This accelerates the overexpression of IL-6 but also results in enhanced tumour necrosis factor-α (TNF-α) and IL-1 concentrations accompanied by a variety of other cytokines and following infiltration of macrophages and neutrophils [15]. SARS-CoV-2 also affects the system by interfering with AngII angiotensin receptor type 1, which also leads to amplification of TNF-α and soluble IL-6 receptors [16]. This activates other hyperinflammatory signal transducers which further reamplify IL-6 following expression of various chemokines including monocyte attracting protein-1 (MCP-1/CCL-2), interleukin-8 (CXCL-8), and IL-6, which provoke infiltration of macrophages and neutrophils into lung tissue [17]. 

**Figure 1:**
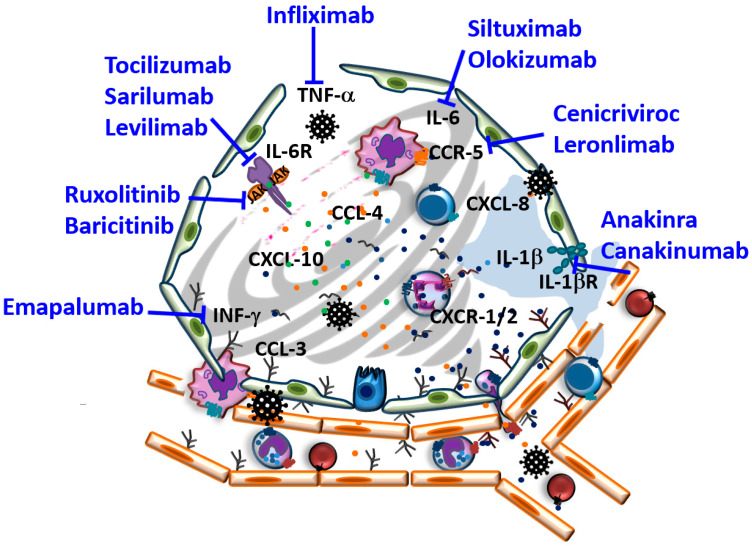
Schematic illustration of severe acute respiratory syndrome coronavirus 2 (SARS-CoV-2) infected alveoli and therapeutically explored targets aiming to interfere with SARS-CoV-2 induced cytokine storm in coronavirus disease (COVID-19) (for further details, see text).

Overacting of early immune response mediated proinflammatory cytokines can result in a cytokine storm (CS, see Table 1 and Figure 1), which is known to be responsible for critical illness, inflammatory failures, multiorgan injury, and thus for mortality in COVID-19 infections. Patients experiencing this phenomenon are known to have a worse prognosis than those who are not affected by hyperinflammatory events and are more likely to develop the CS-induced cytokine release syndrome (CRS) and acute respiratory distress syndrome (ARDS) as a clinical manifestation. ARDS patients encounter hypoxemic respiratory failure accompanied by severe dysfunctions of lung mechanisms, bilateral infiltration on chest images in the absence of cardiac or fluid overload as causative explanations [18,19]. Secreted cytokines from alveolar macrophages such as IL-1, IL-6, CXCL-8 (IL-8), and IL-10, but also TNF-α are responsible for stimulating chemotaxis of neutrophils [20]. Protein-rich edema fluid, infiltrated neutrophils, and platelets are major originators of endothelial and epithelial tissue damage in ARDS as neutrophils accumulate in the microvasculature of lung tissue and, after activation, lead to the release of further pro-inflammatory mediators and toxic, degranulating substances [21,22]. 

Chemokines are considered powerful biomarkers and therapeutic targets in a series of diseases like atherosclerosis, multiple sclerosis, and colitis [23,24,25,26,27,28,29]. Thus, the analysis of serum chemokine and cytokine levels in COVID-19 patients at different severity stages has been considered a diagnostic strategy particularly when correlating their levels with disease progression (Table 1). In early 2020, Huang et al. reported already high plasma levels of the chemokines CXCL-8 (IL-8), CXCL-10 (IP-10), CCL-2 (MCP-1), CCL-3 (MIP-1α), and CCL-4 (MIP-1β) and elevated values for several inflammatory cytokines (IL-1β, IL-1α, IL-7, IL-9, IL-10, FGF, GCSF, GMCSF, IFN-γ, PDGF, TNF- α, and VEGF) in the very first identified COVID-19 patients compared to healthy controls [1]. Xiong et al. added to the list enhanced expression CXCL-1, CXCL-2, and CXCL-6 by performing transcriptome sequencing of RNAs from the bronchoalveolar lavage fluid specimens of patients suffering from COVID-19 in comparison to healthy individuals [30]. Chi, Ge et al. reported increased serum levels of CCL-2, CCL-7 (MCP-3), CXCL-10, CXCL-9 (MIG), and CCL-3 in patients with clinical manifestations. While these data clearly show upregulations of several chemokines, the data for some chemokines like RANTES (Regulated on Activation, Normal T Expressed and Secreted, CCL-5) are less clear: while Zhao Qin et al. [31] demonstrated elevated serum levels in mild but not severe cases of COVID-19, Li et al. [32] described the opposite results.

**Table 1:** Elevated chemokine serum levels in COVID-19 patients Abbreviations: I, increased; ND, no difference to healthy controls.

Chemokine	COVID-19 Severity		Literature
CXCL-8/IL-8	Mild/Moderate/Severe	I	[32,33,34,35,36,37,38,39]
CXCL-9/MIG	Moderate/Severe	I	[37]
CXCL-10/IP-10	Mild/Moderate/Severe	I	[1,29,31,38,39,40,41,42,43]
CCL-2/MCP-1	Mild/Moderate/Severe	I	[1,29,31,40,41,42,43]
CCL-3/MIP-1 alpha	Mild/Moderate/Severe	I	[1,44]
		ND	[40]
CCL-4/MIP-1 beta	Mild/Moderate/Severe	I	[1]
		ND	[40]
CCL-8/MCP-2	Moderate/Severe	I	[42]
CCL-7/MCP-3		I	[42,44]
CCL-5/RANTES	Mild (but not severe)	I	[31]
	Severe (but not mild)	I	[29]
		ND	[37]
	Severe	I	[45]
CCL-11/Eotaxin		ND	[1]

## 2. Clinical-Stage Therapeutics

As a result of COVID-19 emerging as a serious public health issue, there are currently more than 4000 clinical trials of possible treatment with different modes of action and vaccination options ongoing worldwide (see clinicaltrials.gov and www.clinicaltrialsregister.eu). Despite the great amount of evidence of the involvement of chemokines in a plethora of lung inflammatory diseases such asthma, COPD (Chronic obstructive pulmonary disease), cystic fibrosis, and in the inflammatory response that follows lung infectious disease, so far there has been only a limited number of drugs in clinical development that target chemokines either directly or by inhibiting their receptors [46,47] with none having reached the market for these indications. Here, we will focus on clinical-stage therapeutics that bind either directly to chemokines or their receptors or affect chemokine concentrations via precursor regulation (for example, by acting on cytokine and cytokine receptors, as illustrated in Figure 1).

### 2.1. TNF-α-Targeting Therapeutics

TNF-α is one of the most intensively studied pro-inflammatory cytokines and is known to play a crucial role in a plethora of inflammatory diseases. TNF-α fulfils pivotal functions in maintaining the immune system and physiological homeostasis. At the same time, due to their pro-inflammatory properties, TNF-α is also the target for therapeutic antibodies in different diseases like rheumatoid arthritis, Crohn’s disease, and psoriasis [48]. 

It is mainly expressed by macrophages as one of the most abundant mediators in the initial host defence against bacteria, microorganisms, and viruses. TNF-α is highly over-produced in the diseases mentioned above and promotes the expression of several cytokines (including a variety of chemokines), and most abundantly, IL-1 and IL-6 [49]. Thus, TNF-α accelerates the activation of immune cells, which can result in uncontrolled inflammatory response and tissue damage under certain circumstances, like in the case of COVID-19 cytokine storm [50]. Although the five currently approved TNF-α targeting monoclonal antibodies all aim at the same target, their molecular mechanisms are different. Infliximab (chimeric mouse/human), adalimumab, and golimumab (both fully human) are three IgG1 monoclonal antibodies that bind to soluble and transmembrane forms of TNF-α with high affinity, preventing TNF-α from interacting with its receptors. Etanercept is a chimeric fusion protein that consists of the p75 part of TNFR2 and a human IgG1 Fc domain. Finally, certolizumab, a PEGylated humanised Fab fragment, can block TNF- α in both its soluble and transmembrane forms [51]. 

Even knowing that TNF-α is a decisive factor in the emergence and progression of the life-threatening cytokine storm in COVID-19 patients, the specific role of TNF-α in SARS-CoV-2 infections is not yet clarified. However, elevated serum levels have been reported in hospitalised COVID-19 patients with severe acute respiratory syndrome [1]. Results from the SECURE (Surveillance Epidemiology of Coronavirus Under Research Exclusion) IBD database reported that IBD patients under treatment with anti-TNF-α therapy who were diagnosed with COVID-19 showed equal or even better clinical outcomes than patients without prior treatment with the TNF-α -blocking drugs [52]. These results are supported by the similar outcomes observed for an ulcerative colitis patient (receiving infliximab) who was tested positive for coronavirus and developed mild COVID-19 symptoms, resolving after only one week [53]. Hence, the potential of anti-TNF-α drugs is of greatest interest, and their use in hospitalised COVID-19 patients is highly discussed as a possibly very effective treatment option, especially if given in the early stage of the disease.

In a retrospective study, Stallmach et al. reported rapid mitigation of severe COVID-19 patients treated with infliximab, compared to a control COVID-19 group, who only received the best standard therapy [54]. Infliximab is currently tested in a randomised phase 3 clinical trial among other immune-modulating candidates for treating COVID-19 as an add-on therapy to the already approved remdesivir (Gilead Sciences, Inc., Foster City, CA, USA) (NCT04593940). Also, adalimumab (AbbVie Inc., Lake Bluff, IL, USA) is currently being tested for its activity in reducing symptoms of COVID-19 patients at the Oxford Clinical Trials Research Unit in a trial started in July this year [55]. 

### 2.2. Interleukin-1-Targeting Therapeutics

Since SARS-CoV-2 can induce the production of IL-1β, different IL-1β, and IL-1β receptors antagonising therapeutics have been tested in hospitalised COVID-19 patients with ARDS in the attempt to reduce the hyperinflammatory CRS. Anakinra (Swedish Orphan Biovitrum, Stockholm, Sweden), a recombinant IL-1β receptor antagonist, is currently under investigation in more than 20 clinical trials to reduce cytokine-induced multiorgan and particularly pulmonary failures. Prior studies in a small number of patients showed mixed results. The first study with anakinra in severely ill COVID-19 patients suffering from ARDS was conducted in May 2020 by Cavalli et al. that reported a significant decrease of mortality and an overall improvement of respiratory functions in anakinra plus 4-aminoquinoline-treated cases compared to controls on SOC (standard of care) [56]. These results were corroborated by several other controlled studies with small patient numbers performed all around the world, which reported a favourable response to anakinra treatment [57,58,59,60,61,62]. The most extensive, currently still recruiting study is an open-ended clinical trial with a target of 7100 patients showing mild to severe COVID-19 symptoms (NCT02735707, EudraCT2015-002340-14). There, anakinra is tested for its efficiency among a panel of other sutherapeutics. The activity of anakinra against COVID-19 is also phase 3 tested in combination therapies with Janus kinases (JAKs) inhibiting ruxolitinib (Incyte Corp in the US, Novartis rest of the world) (NCT04424056, EudraCT2020-001754-21) and with IL-6/receptor-axis interfering agents siltuximab (Janssen Pharmaceutica Beerse, Belgium) and tocilizumab (Hoffmann-La Roche, Basel, Switzerland) (NCT04330638). Since results from extensive trials are still pending, anakinra has yet no approval for use in COVID-19. 

Another IL-1 blocker, canakinumab (Novartis, Basel, Switzerland) a human anti-IL-1β monoclonal antibody currently in use for the treatment of different autoimmune disorders, is also tested in COVID-19-associated lung failures in five active studies according to clinicaltrials.gov [62]. Preliminary data show a rapid and significant reduction in the overall inflammatory markers [63] and therefore warrants further investigation as a potential treatment option in COVID-19.

### 2.3. Interleukin-6 and Interleukin-6-Receptor-Targeting Therapeutics

IL-6 is a major key player in the early immune response to infections and also mediates the uncontrolled production and release of chemokines and cytokines [64]. The cytokine is produced by CD14+CD16+ monocytes following IL-1β and TNF-α stimulation. Highly increased IL-6 concentrations can be decisive for severe inflammatory conditions in COVID-19. Serum IL-6 levels have been found to be significantly increased in critically ill patients; compared to moderate and severe COVID-19 cases, its levels show direct correlation to the disease severities [65,66,67]. Tocilizumab (TCZ; Hoffmann-La Roche, Basel, Switzerland), an anti-human IL-6 receptor humanised monoclonal IgG1 antibody, has gained great attention because it is supposed to interfere with the cytokine-mediated overproduction of pro-inflammatory cytokines in COVID-19 patients who suffer from cytokine storm-related malfunctions, such as multiple organ dysfunction and cardiovascular collapses. Tocilizumab binds soluble and membrane-bound IL-6 receptors and thus inhibits IL-6 binding and further blocks its signalling with the IL-6 receptor-dependent cells: B and T lymphocytes, neutrophils, eosinophils, and basophils. TCZ is already used in the treatment of rheumatoid and systemic juvenile idiopathic arthritis, but it is also FDA approved for use in cytokine release syndrome (CRS) caused by CAR-T (Chimeric antigen receptor T) cells [68]. Its high level of safety has been demonstrated in the course of testing for the aforementioned indications, which is the reason it was possible to test it as one of the first candidates in severe and critical COVID-19 patients in recent months [69,70]. Tocilizumab is one of the most tested compounds among the immune-modulating drugs (as can be seen in Table 2). Nevertheless, the inconsistency of the results does not allow any conclusion—mainly due to the very low number of patients [71,72]. Although not always reaching statistical significance, almost all studies showed improvements in survival rate, respiratory functions, reduction in ICU admission, and reduced the need for mechanical ventilation in COVID-19 patients with rather severe symptoms when treated with TCZ in addition to standard of care [73,74,75,76,77,78,79,80,81,82,83,84]. However, in a randomised, double-blind, placebo-controlled clinical trial, tocilizumab did not show any beneficial effects in terms of mortality rate or preventing intubation in moderately ill, hospitalised COVID-19 patients [85]. Thus, new clinical studies testing TCZ in COVID-19 patients need to be extended in cohort numbers, defining the laboratory parameters for disease severity and with refinement of the treatment duration and dosages. 

Other therapeutics interfering in the IL-6 pathways are also being tested for their efficacy in COVID-19. The monoclonal IgG1 anti-IL-6 receptor antibody sarilumab, which is approved for the treatment of rheumatoid arthritis in patients who do not respond to at least one disease-modifying anti-rheumatic drug, is currently under investigation. It shares the same mode of action as the previously described tocilizumab but binds the IL-6 receptor with higher affinity [86]. Gremese et al. showed a trend of sarilumab to improve the clinical outcome in severe cases of COVID-19, compared to SOC-treated patients, albeit with only 53 participants in the study [87]. This result was supported by other early studies with limited patients numbers, which suggested that sarilumab-mediated IL-6 reduction correlated with faster recovery [88,89,90]. However, in September 2020, Sanofi pharma’s phase 3 randomised study including 420 severely or critically ill COVID-19 patients (NCT04327388) with their sarilumab-product Kevzara^®^ (Regeneron Pharmaceuticals Tarrytown, NY, USA and Sanofi, Paris, France) did not meet the defined endpoints, and the company does not anticipate any further research on sarilumab in COVID-19.

Russia has already approved the fully human monoclonal anti-IL-6 receptor antibody (for both soluble and membrane-bound receptors) levilimab for use in COVID-19 patients with severe respiratory symptoms. The antibody which was initially developed for use in the treatment of rheumatoid arthritis by BIOCAD (Sankt-Peterburg, Russia) has received fast-track state approval and is currently under further phase 3 clinical trial NCT04397562. The earlier results from phase 1 and 2 showed significantly reduced mortality in levilimab-treated COVID-19 patients.

Another possible therapeutic route to interfere with the IL-6 mediated cytokine enhancing signalling is represented by siltuximab, a recombinant human–mouse chimeric anti-IL-6 antibody, which is currently approved by the FDA (Food and Drug Administration) and EMA (European Medicines Agency) for the treatment of multicentric Castleman’s disease (MCD) [91]. It can prevent IL-6 binding to both soluble and membrane-bound IL-6 receptors and further inhibits IL-6 signalling transduction. EUSA pharma (Hemel Hempstead, United Kingdom) conducted an observational clinical study (Siltuximab in severe COVID-19, NCT04322188) with its MCD drug Sylvant^®^ on 30 patients with severe disease form who received the drug as either single or double administration [92]. The primary outcome of a lower 30-day mortality rate in siltuximab-treated patients compared to patients who only received the best supportive care was met. This finding set siltuximab as a potentially potent treatment and warrants confirmation by well-controlled randomised studies. Thus, siltuximab is currently tested in multiple clinical trials and also in one phase 3 trial (NCT04330638, phase 3). In this study, individual or combined treatment with siltuximab, anakinra, and tocilizumab are being evaluated in terms of safety and efficacy.

Another IL-6 antibody called olokizumab (R-Pharma) has currently joined the race due to its very encouraging effects in the treatment of SARS-CoV-2-infected, hospitalised patients who show mild or moderate symptoms. It is currently in a phase 2/3 clinical trial (NCT04452474).

### 2.4. Interfering on IP-10/CXCL-10 Pathway with IFNγ-Targeting Compounds

As can be seen in Table 1, several studies report elevated IP-10 (CXCL-10) levels in COVID-19 patients. IP-10 has been found to correlate significantly with the severity level of COVID-19 patients, with values only slightly increased in asymptomatic cases but highly elevated in moderate and critically ill patients—thus suggesting CXCL-10 as an independent predictor for COVID-19 progression [39,40,44]. The contribution of CXCL-10 to the disease progression makes it a potential putative target for inhibiting cytokine storm-related excessive inflammatory events. Also responsible for the original name, IP-10 (IFN-γ-inducible protein) expression is primarily induced by interferon-γ (IFN-γ). After binding the IFN γ receptors 1 and 2, the JAK-STAT pathways get activated, which results in the secretion of various cytokines and chemokines, including CXCL10. When considering the pathological increased serum CXCL-10 levels reported in COVID-19 patients, therapeutics inhibiting these axes by IFN-γ antagonism represent a very promising strategy. The monoclonal anti-IFN-γ-antibody emapalumab (Swedish Orphan Biovitrum AB, Stockholm, Sweden) is an FDA-approved first-in-class medication for the treatment of primary hemophagocytic lymphohistiocytosis, which is also characterised by hypercytokinemia [93]. Emapalumab is currently tested in a phase 2/3 multicentre study (NCT04324021, EudraCT2020-001167-93) where patients with respiratory distress failure receive every third day 6 mg/kg emapalumab i.v. for a total of five infusions as an add-on therapy to SOC. In the course of this three-armed trial, anakinra is also tested, and both treatments are compared to SOC. Clinical data from these trials will show if emapalumab will be able to improve outcomes in SARS-CoV-2 infected patients.

### 2.5. JAK Inhibitors

Another currently investigated strategy for reducing the cytokine-induced hyperinflammatory response in the lung is the inhibition of Janus kinases (JAKs). JAKs are used as intracellular signalling tranductors for Type I and Type II cytokine receptors. Moreover, JAK inhibitors have demonstrated a dual activity profile showing both anti-viral and anti-inflammatory features which makes them very interesting targets for COVID-19 patients [94,95]. Ruxolitinib (Incyte Corp in the US and Novartis), currently EMA and FDA approved for the treatment of rheumatoid arthritis, and its structural analogue baricitinib (Eli Lilly) approved for myeloproliferative neoplasms, are two representatives of JAK-antagonizing biologics, both blocking Janus kinases 1 and 2. Baricitnib has been shown to also prevent SARS-CoV-2 entry by inhibiting IFNα2-mediated ACE2 expression in 3D cultures of primary human liver cells, which suggests possible activity against SARS-CoV-2 liver infectivity [96]. Data published earlier this year show beneficial effects of ruxolitinib in COVID-19 patients with severe pulmonary inflammations [97,98,99]. A broad panel of multicentre clinical trials have been initiated with JAK inhibitor therapy alone, or in combinations with other drugs such as simvastatin, anakinra, and tocilizumab (Table 2).

### 2.6. CCR5-Antagonizing Therapeutics

Chemokine receptor 5 (CCR5) contains seven transmembrane domains and is expressed by various tissues, including endothelial and epithelial cells, as well as in vascular smooth muscle and fibroblasts [100]. Since 1996, CCR5 is known to be an important co-receptor for human immunodeficiency virus type 1 (HIV-1) to enter specific target immune cells, since CCR5 ligands were able to inhibit the viral entry into CD4+ cells and have repeatedly been at the centre of anti-viral questions since then [101]. Although ACE2 receptor has been identified as the crucial key for SARS-CoV-2 to enter target host cells, CCR5 may also be involved. Its chemokine ligands CCL-5 (RANTES), CCL-3, CCL-4 (also known as MIP-1α and 1β, respectively), and CCL-3L1 are upregulated in severe COVID-19 cases [45]. 

The CCR5 receptor antagonist maraviroc (Pfizer, UK), represents a selective, slowly reversible, small molecule antagonist of the interaction between human CCR5 and HIV-1 gp120. Blocking this interaction prevents CCR5-tropic HIV-1 entry into the cell [102]. Outside of its efficacy in blocking CCR5-tropic HIV-1 entry, CCR5 antagonism could be useful in preventing the ‘second wave’ of inflammatory mediators in SARS-CoV-2 patients that are responsible for the acute respiratory distress syndrome (ARDS). Indeed, several studies looking at both gene expression in lungs and blood cytokines and chemokines levels have related chemokine signalling clusters with COVID-19 severity, and among these CCR5 ligands [13,103]. Moreover, SARS-CoV infected airway epithelial cells and monocyte-derived dendritic cells express high levels of CCL-5 [104,105], and it seems possible that similarly high levels of expression could be present in patients affected by SARS-CoV-2. Disruption of the CCL-5-CCR5 axis therefore seems an attractive therapeutic approach. An open-label study (phase 2) is currently ongoing to establish whether one week of treatment with maraviroc, used at the approved dosages for HIV, is safe and tolerable in a total of 16 patients with moderate and severe SARS-CoV-2. Additionally, assessment of clinical improvement and changes in CCL-5, IL-6, and Chitinase 3-like-protein-1 levels at day 7 will be analysed (NCT04435522). Another CCR5 antagonist named Cenicriviroc (CVC; Takeda and Tobira Therapeutics) was also initially used for the inhibition of HIV replication, and it has become a very promising candidate for the treatment of COVID-19 patients by preventing SARS-CoV-2 replication. Small molecule CVC acts as a CCR5 antagonist and additionally inhibits the binding of CCL-2 to CCR2b expressing cells. One trial is already in phase 3 of clinical investigations (NCT04593940) where CVC is tested among three other immune-modulating agents against COVID-19.

Another CCR5-targeting, viral-entry inhibiting drug, which is currently investigated in its efficiency to reduce COVID-19 symptoms, is the monoclonal IgG4 antibody leronlimab (CytoDyn, Vancouver, WA, Canada), formerly PRO140. Previously performed but rather small in sample number and not placebo-controlled studies suggested the beneficial effect of leronlimab induced decrease of IL-6 levels in severe COVID-19: Over the two-week study period 6/10 patients survived, two were extubated, and one patient was discharged. Complete CCR5 receptor occupancy in all patients was observed by day 7 and a statistically significant reduction of plasma IL-6 compared to baseline, restoration of the CD4/CD8 ratio, and resolution of SARS-CoV2 plasma viremia compared to controls [45,106,107]. Leronlimab is now tested in two phase 2 clinical trials—NCT04347239 and NCT04343651. The results from these studies will clarify if antagonism of the CCR5 receptor via small molecule antagonist like maraviroc or using a CCR5-specific human IgG4 monoclonal antibody-like leronlimab may be a viable way to improve the clinical outcome and survival of COVID-19 patients.

### 2.7. Targeting the CXCL-8 Pathway—A Promising Approach?

When analysing serum levels of hospitalised COVID-19 patients, Del Valle et al. reported increased serum levels of IL-6, TNF- α, and CXCL-8 (IL-8) [35]. When considering the often occurring neutrophilia in SARS-CoV-2 infected patients, the role of CXCL-8 needs to be further investigated because it is a crucial factor in neutrophil migration [108]. Therefore, the University of Columbia started a phase 2 first-in-human study for testing a neutralising IL-8 antibody with BMS-986253 (Bristol-Myers-Squibb, New York, USA) in a single centre trial with hospitalised COVID-19 patients (NCT04347226), of which the primary outcome data will be available in September 2021. 

Several small molecules that are antagonists of the CXCL8 receptors CXCR-2, like AZD506 (AstraZeneca), navarixin, or danirixin, have been in clinical trials in several lung diseases like COPD or asthma bronchiale. However, so far none of them have been tested in COVID-19 patients.

## 3. Conclusions

Coronavirus disease (COVID-19), as triggered by an infection with severe acute respiratory syndrome coronavirus 2 (SARS-CoV-2), has caused an unprecedented pandemic crisis. The fast-spreading disease has worldwide consequences beyond medical issues which drives scientists around the globe to not only work hard on the development of vaccines to control the spread of the disease but also find effective therapeutic treatment options for mild and/or severely ill COVID-19 patients.

One of the most dangerous complications in COVID-19 patients is the acute respiratory distress syndrome (ARDS), which is associated with a hyperactivated immune system and high serum levels of chemokines and other cytokines referred to the term cytokine storm.

We have reviewed currently investigated therapeutic strategies that interfere with the hyperinflammatory pathways by blocking different chemokines, cytokines, and their corresponding receptors. The initially performed clinical studies were conducted in an exploratory way in small numbers of patients who were already critically ill with rather severe symptoms and high risk of death and often under individual compassionate care. Most of the tested candidates are anti-inflammatory monoclonal antibodies on the market for the treatment of chronic diseases like rheumatoid arthritis. Data from properly regulated, double-blinded, placebo-controlled clinical trials in large numbers of COVID-19 patients are still missing for most of the therapeutics we have presented here. Moreover, comprehensive time-course evaluations regarding systemic levels of the inflammatory cytokines/chemokines from COVID-19 patients with different disease progression are still required to validate any of these molecules as useful biomarkers for COVID-19. A more comprehensive picture will result from such kinetic analyses and will allow for a better design of future clinical trials. Especially for testing potential therapeutics following COVID-19 infections, it is crucial to identify the stage of disease progression at which treatment with a certain drug is most effective. This is particularly important for chemokine-targeting approaches since the release of chemokines is precisely timed with the physiological need for certain immune cells, a balance which shifts during the progression of COVID-19 infection resulting in the worst case in a cytokine storm with all its consequences. 

IL-6 and IL-6 receptor-blocking drugs showed mixed but predominantly promising results in patients suffering from ARDS. Based on the number of placebo-controlled randomised studies, tocilizumab seems to be the most promising candidate among this class. Nevertheless, IL-6-blocking substances must not be seen as the final key treatments to combat COVID-19, as IL-6 is not solely responsible for the cytokine storm in COVID-19 because it still remains unclear whether elevated IL-6 in viral infections, and SARS-CoV-2 in particular, represents a therapeutic target or part of a functioning adaptive immune response. Indeed, studies have shown that intravenous administration of monoclonal IL-6 in healthy volunteers, at levels significantly exceeding the concentrations found in COVID-19, did not result in pulmonary adverse events, which further questions the importance of IL-6 in COVID-19 [109,110]. Moreover, patients with severe COVID-19 who have been administered anti–IL-6 treatments have reported having higher numbers of secondary infections [111]. All mentioned substances, except sarilumab, are still in ongoing clinical trials to evaluate their safety and efficacy on different aspects of COVID-19 outcomes. Possible side-effects should also be considered for other compounds: the general toxicity profiles of anti-TNF alpha drugs enhancing the risk for bacterial, fungal, and virus infection. However, no evidence was found in the above-discussed studies that TNF alpha-blocking agents are increasing the number of infections. Furthermore, although JAK-inhibiting ruxolitinib is known to be well tolerated with low toxicities, the prothrombic risk is a class effect of JAK inhibitors and must be considered when using it as an off-label therapeutic.

A very interesting molecular interface with high therapeutic potential in the future are the glycosaminoglycans (GAGs) because they bind and present chemokines on the one hand and act as co-receptors for SARS-CoV-2 on the other hand. Thus, molecules which interfere with chemokine binding to GAGs and simultaneously inhibit SARS-CoV-2 spike protein interacting with GAGs may pose a bi-functional approach to inhibit COVID-19 progression on an immune cell level combined with inhibition of viral entry. 

## Figures and Tables

**Table 2:** Summary of selected therapeutic strategies targeting the cytokine storm in COVID-19 in phase 3 and 4 clinical trials registered on clinicaltrials.gov and clinicaltrialsregister.eu databases Abbreviations: SOC, standard of care; IMV, invasive mechanical ventilation; MV, mechanical ventilation; ICU, intensive care unit; ECMO, extracorporeal membrane oxygenation.

Drug Class	Name	Database Number	Phase	Status	Treatment Regimen	Number of Patients	Primary Outcomes
**TNF-α**	**Infliximab**	NCT04593940	3	Recruiting	Infliximab (5 mg/kg I,v, single dose infusion on day 1) + Remdesivir (SOC)	*n* = 2160	Time to recovery by day 29
**IL-1 receptor** **Inhibitors**	**Anakinra**	NCT04424056 EudraCT2020-001754-21	3	Not yet recruiting	Anakinra +/− Ruxolitinib (Stages 2b/3) Anakinra and Ruxolitinib (Advanced stage 3)	*n* = 366	Ventilation free days by day 28
		NCT04364009 EudraCT2020-001734-36	3	Suspended (efficacy and safety reasons)	400 mg from day 1 to day 3 (2 × 100 mg every 12 h) and 200 mg the remaining 7 days	*n* = 311	Patients alive and not requiring IMV or ECMO at Day 14
		NCT04443881	2|3	Recruiting	100 mg every 6 h i.v. infusion up to 4 times a day for a maximum of 15 days	*n* = 180	Patients nr not requiring MV by day 15; time to MV, time to oxygen saturation normalization, length ICU and hospitalization
		NCT04362111	3	Recruiting	Anakinra 100 mg s.c. every 6 h for 10 days (decreased to twice a day for subjects meeting complete response criteria at day 5)	*n* = 30	Percentage of patients discharged from hospital w/o need for intubation or MV
		NCT04324021 EudraCT2020-001167-93	2|3	Recruiting	100 mg i.v. every 6 h for 15 days	*n* = 54	Proportion of patients not requiring MV or ECMO
		NCT04330638	3	Recruiting	Anakinra 100 mg s.c. for 28 days (or until hospital discharge) Anakinra (100 mg s.c. 28 days) + Siltuximab (single i.v. infusion 11 mg/kg) Anakinra (100 mg s.c. 28 days) + Tocilizumab (single i.v. infusion 8 mg/kg max. 800 mg/injection)	*n* = 342	Time to an improvement of two points on a six-category ordinal scale or discharge from hospital
		NCT02735707 EudraCT2015-002340-14	4	Recruiting	300 mg as bolus via central or peripheral line, 100 mg every 6 h i.v. administration	*n* = 1100	All-cause mortality to day 90 Days alive and not receiving organ support in ICU after 21 days
	**Canakinumab**	NCT04362813 EudraCT2020-001370-30	3	Active, not recruiting	450 mg (BW 40–60 kg), 600 mg (BW 60–80 kg), 750 mg (BW > 80 kg) in 250 mL 5% dextrose i.v. infusion over 2 h; single dose	*n* = 451	Number of patients with survival without requiring IMV from day 3 to day 29
**IL-6 Receptor** **Antagonists**	**Tocilizumab**	NCT04377750	4	Recruiting	8 mg/kg (up to a maximum of 800 mg) i.v. infusion	*n* = 500	One-month mortality rate
		NCT02735707 EudraCT015-002340-14	4	Recruiting Ongoing	8 mg/kg (up to a maximum of 800 mg) i.v. infusion; single dose	*n* = 7100 *n* = 4000	All-cause mortality rate by day 90, days alive and not requiring ICU
		NCT04577534 EudraCT2020-002039-31	3	Recruiting Ongoing	i.v. infusion according to weight of patient	*n* = 90	Clinical status at day 28 (assessed using 7-category ordinary scale)
		NCT04412772	3	Recruiting	8 mg/kg (up to a maximum of 800 mg) i.v. infusion; up to one additional dose	*n* = 300	Clinical status at day 28 (assessed using 7-category ordinary scale)
		NCT04372186	3	Active, not recruiting	8 mg/kg (up to a maximum of 800 mg) i.v. infusion	*n* = 379	Cumulative Proportion of Participant requiring MV by day 28
		NCT04356937	3	Active, not recruiting	8 mg/kg (up to a maximum of 800 mg) i.v. infusion	*n* = 243	Time to requiring MV and intubation or death
		NCT04320615 EudraCT2020-001154-22	3	Completed Completed/ongoing	8 mg/kg (up to a maximum of 800 mg) i.v. infusion	*n* = 450	Clinical Status Assessed Using a 7-Category Ordinal Scale at day 28
		NCT04424056 EudraCT2020-001754-21	3	Not yet recruiting	Tocilizumab Tocilizumab + Ruxolitinib	*n* = 366	Number of days living without MV at day 28
		NCT04409262	3	Recruiting	Tocilizumab i.v. infusion on day 1 + Remdesivir for 10 days	*n* = 500	Time to hospital discharge by day 28
		NCT04361032	3	Not yet recruiting	8 mg/kg injection by day 1	*n* = 260	Mortality rate at 90 days
		NCT04403685	3	Terminated	8 mg/kg (up to a maximum of 800 mg) i.v. infusion; single dose	*n* = 129	Clinical status after 15 days by 7-points ordinal scale
		NCT04423042	3	Not yet recruiting	8 mg/kg (up to a maximum of 800 mg) i.v. infusion; single dose (possible repetition within 12–28 h)	*n* = 30	All-cause mortality after 30 days
		NCT04330638	3	Recruiting	8 mg/kg (up to a maximum of 800 mg) i.v. infusion; single dose	*n* = 342	Time to clinical improvement of two points on a six-category ordinal scale or hospital discharge
		EudraCT2020-001500-41	3	Ongoing	i.v. Solutions; 20 mg/mL	*n* = 342	Time to an improvement of two points on a six-category ordinal scale or hospital discharge
		NCT04381936 EudraCT2020-001113-21	2|3	Recruiting	20 mg/mL vs hydrocortisone; methylprednisolone; immunoglibuline, AS13; aspirine; colchicine or placebo	*n* = 20,000	All-cause mortality after 28 days, peadietric population
		NCT04349410	2|3	Completed	8 mg/kg (up to a maximum of 800 mg) i.v. infusion over 1 h; single dose	*n* = 1800	Tissue improvement measured by Fleming Method for Tissue and Vascular Differentiation and Metabolism (FMTVDM)
		EudraCT2020-002275-34	4	Ongoing	i.v. infusion; 800–1200 mg;	*n* = 290	Mortality at day 28 after treatment initiation
		EudraCT2020-001854-23	2|3	Ongoing	Solution for injection; 162 mg/mL; equal	*n* = 1400	Proportion of patients with PaO_2_/FiO_2_ ratio <200 mmHg at day 10
		EudraCT2020-001707-16	3	Ongoing	i.v. infusion; 20 mg/mL	*n* = 60	Respiratory situation at 24 h, day 3 and day 7 (PaO_2_/FiO_2_ ratio)
		EudraCT2020-001246-18	2|3	Ongoing	i.v. infusion; 20 mg/mL; 20 mL	*n* = 1000	ICU requiring group: Cumulative incidence of successful tracheal extubation at day 14, OMS progression scale >7 at day 4 Non-ICU requiring group: survival rate without MV at day 14, OMS progression scale ≤5
	**Sarilumab**	NCT04315298	3	Completed	i.v.; single or multiple doses	*n* = 1912	Proportion of patients with clinical improvement of 2 points out of a 7-point category ordinal scale at day 22
		NCT04341870 (DSMB recommendation (futility)	2|3	Suspended	Sarilumab 400 mg i.v. over 1 h, single dose Sarilumab (400 mg/1h i.v.) + Azithromycin (oral 500 mg day 1, 250 mg day 2 to day 5) + Hydroxychloroquin (oral 600 mg day 1 to day 10)	*n* = 27	Need for ventilation (invasive and non- invasive), ICU or death over 14 days
		NCT04327388 EudraCT 2020-001162-12	3	Completed	Variante A:1 dose i.v. on day 1, 2nd dose after 24 or 48 h if required Variant B: 2 doses i.v. on day 1, further 2 doses after 24 or 48 h if required i.v. 200 mg; equal	*n* = 860	Time of clinical improvement (2 points out of a 7-point category ordinal scale) after 29 days
		NCT04324073	2|3	Active, not recruiting	400 mg for 1 h i.v. infusion at day 1	*n* = 239	survival rate without need of ventilation, WHO progression scale ≥5 at day4 for patients with NI ventilation at day 4, Cumulative incidence of successful tracheal extubation (<48 h) at day 14, WHO progression scale at day 4
		EudraCT2020-001246-18	2|3	Ongoing	s.c. 200 mg	*n* = 1000	ICU requiring group: Cumulative incidence of successful tracheal extubation at day 14, OMS progression scale >7 at day 4 Non-ICU requiring group: survival rate without MV at day 14, OMS progression scale ≤5
		NCT02735707 EudraCT2015-002340-14	4	Recruiting	400 mg i.v. over 1 h; single dose	*n* = 1100	All-cause mortality to day 90 Days alive and not receiving organ support in ICU after 21 days
		EudraCT2020-001390-76	3	Ongoing	i.v. 400 mg	*n* = 171	Time of clinical improvement (2 points out of a 7-point category ordinal scale)
		EudraCT2020-001162-12	2|3	Ongoing	i.v. 200 mg	*n* = 440	Time of clinical improvement (2 points out of a 7-point category ordinal scale)
		EudraCT2020-001290-74		Ongoing	i.v. 200 mg	*n* = 216	Time of clinical improvement (2 points out of a 7-point category ordinal scale)
		EudraCT 2020-001854-23	2|3	ongoing	200 mg tablet for oral use (in addition to hydroxychloroquin)	*n* = 1400	PaO_2_/FiO_2_ ratio <200 mmHg
	**Levilimab**	NCT04397562	3	Completed	s.c. 324 mg	*n* = 206	Proportion of patients with improvement of 2 points out of a 7-point category ordinal scale
**IL-6 Inhibitors**	**Siltuximab**	NCT04330638 EudraCT2020-001500-41	3	Recruiting ongoing	i.v. 11 mg/kg i.v. 11 mg/kg single dose+ Anakinra s.c. 100 mg for 28 days i.v. 400 mg	*n* = 342	Time to clinical improvement (2 points out of a 6-point category ordinal scale)
	**Olokizumab**	NCT04452474	2|3	Not yet recruiting	s.c. 64 mg (single injection Olokizumab)+SOC	*n* = 376	Time to clinical improvement (2 points out of a 5-point category ordinal scale)
**IFNγ Inhibitor**	**Emapalumab**	NCT04324021 EudraCT2020-001167-93	2|3	Recruiting	6 mg/kg (day1) and 3 mg/kg (day 4,7,10 and 13) i.v. infusions	*n* = 54	proportion of patients not requiring IMV or ECMO
**JAK Inhibitors**	**Ruxolitinib**	NCT04377620	3	Recruiting	Variant A: 5 mg administered BID 12 h apart Variant B: 15 mg administered BID 12 h apart	*n* = 500	Mortality rate by day 29
		NCT04362137 EudraCT2020-001662-11	3	Completed	5 mg tablets twice a day (BID) for 14 (or 28) days	*n* = 834	Proportion of patients who die, develop respiratory failure or require ICU by day 29
		NCT04348071	2|3	Not yet recruiting	10 mg twice daily for 14 days	*n* = 80	*For Phase 3*: Percentage of patients reporting each severity on an 8-point ordinal scale at Day 15
		NCT04477993	2|3	Recruiting	5 mg p.o. BID for 14 days	*n* = 200	Composite Outcome of death or ICU admission or MV at day 14
		EudraCT 2020-001777-71	4	Ongoing	oral administration of tablets	*n* = 59	Number requiring MV or death by day 28
	**Baricitinib**	NCT04421027 EudraCT 2020-001517-21	3	Recruiting Ongoing	4 mg oral dose 2 mg oral	*n* = 1600	Percentage of Patients who die or require non-invasive ventilation(High-Flow Oxygen or IMV including ECMO
		NCT04340232	2|3	Not yet recruiting	2 mg oral dose daily for 14 days	*n* = 80	*For Phase 3*: Percentage of patients reporting each severity on an 8-point ordinal scale at Day 15
		NCT04390464 EudraCT 2020-001354-22	4	Recruiting Ongoing	4mg oral dose daily for 14 days 2–4 mg oral dose	*n* = 1167	Time to reach death, MV, ECMO, cardiovascular organ support or renal failure
		NCT04358614	2|3	Completed	4 mg oral dose daily for 14 days	*n* = 12	All adverse events
		NCT04320277	2|3	Not yet recruiting	4mg oral dose daily (morning) for 14 days	*n* = 200	Percentage of patients requiring ICU admission
		NCT04401579	3	Active, not recruiting	Baricitinib (2 × 2 mg tablets, orally, for 14 days) + Remdesevir (200 mg i.v. on day 1, 100 mg once-daily maintenance up to day 10)	*n* = 1034	Time to recovery (reaching one out of three categories from ordinal scale) for 29 days
		NCT04640168 EudraCT2020-001052-18	3	Not yet recruiting Ongoing	Baricitinib (2 × 2 mg orally daily for 14 days) + Remdesevir as SOC (200 mg i.v.)	*n* = 2300	Proportion of patients not meeting death, IMV or ECMO
		EudraCT2020-001246-18	2|3	Ongoing	2 mg tablet for oral use	*n* = 1000	ICU requiring group: Cumulative incidence of successful tracheal extubation at day 14, OMS progression scale >7 at day 4 Non-ICU requiring group: survival rate without MV at day 14, OMS progression scale ≤5
		EudraCT2020-001854-23	2|3	Ongoing	2 mg and 4 mg tablets for oral use	*n* = 1400	Proportion of patients with PaO_2_/FiO_2_ <200 mm Hg
		EudraCT2020-001367-88	3	Prematurely Ended	4 mg (2 mg 2 tablets) daily for 7 days	*n* = 6	All-cause mortality or need for IMV by day 28
**CCR5 Antagonists**	**Cenicriviroc**	NCT04593940	3	Recruiting	Cenicriviroc (day 1: 300 mg morning + 150 mg evening; day 2 to day 29: 300 mg) + Remdesevir (for SOC)	*n* = 2160	Time to recovery by day 29
	**Leronlimab**	EudraCT2020-001996-33	2b|3	Ongoing	s.c. injection 170 mg/mL (PRO140)	*n* = 290	All-cause mortality at day 28

## Data Availability

There are no further data available.

## References

[1] Huang C., Wang Y., Li X., Ren L., Zhao J., Hu Y., Zhang L., Fan G., Xu J., Gu X. (2020). Clinical features of patients infected with 2019 novel coronavirus in Wuhan, China. Lancet.

[2] Yang J., Zheng Y., Gou X., Pu K., Chen Z., Guo Q., Ji R., Wang H., Wang Y., Zhou Y. (2020). Prevalence of comorbidities and its effects in patients infected with SARS-CoV-2: A systematic review and meta-analysis. Int. J. Infect. Dis..

[3] Callender L.A., Curran M., Bates S.M., Mairesse M., Weigandt J., Betts C.J. (2020). The Impact of Pre-existing Comorbidities and Therapeutic Interventions on COVID-19. Front. Immunol..

[4] Buitrago-Garcia D., Egli-Gany D., Counotte M.J., Hossmann S., Imeri H., Ipekci A.M., Salanti G., Low N. (2020). Occurrence and transmission potential of asymptomatic and presymptomatic SARS-CoV-2 infections: A living systematic review and meta-analysis. PLoS Med..

[5] Guan W.-J., Ni Z.-Y., Hu Y., Liang W.H., Ou C.Q., He J.X., Liu L., Shan H., Lei C.L., Hui D.S.C. (2020). Clinical Characteristics of Coronavirus Disease 2019 in China. N. Engl. J. Med..

[6] U.S. Food and Drug Administration Coronavirus COVID-19 Update FDA Authorizes Monoclonal Antibodies Treatment COVID-19). https://www.fda.gov/news-events/press-announcements/coronavirus-covid-19-update-fda-authorizes-monoclonal-antibodies-treatment-covid-19.

[7] Lau S.K.P., Lau C.C.Y., Chan K.-H., Li C.P.Y., Chen H., Jin D.-Y., Chan J.F.W., Woo P.C.Y., Yuen K.-Y. (2013). Delayed induction of proinflammatory cytokines and suppression of innate antiviral response by the novel Middle East respiratory syndrome coronavirus: Implications for pathogenesis and treatment. J. Gen. Virol..

[8] Kim E.S., Choe P.G., Park W.B., Oh H.S., Kim E.J., Nam E.Y., Na S.H., Kim M., Song K.-H., Bang J.H. (2016). Clinical Progression and Cytokine Profiles of Middle East Respiratory Syndrome Coronavirus Infection. J. Korean Med. Sci..

[9] Rot A., von Andrian U.H. (2004). Chemokines in innate and adaptive host defense: Basic chemokinese grammar for immune cells. Annu. Rev. Immunol..

[10] Tanaka Y., Adams D.H., Shaw S. (1993). Proteoglycans on endothelial cells present adhesion-inducing cytokines to leukocytes. Immunol. Today.

[11] Puneet P., Moochhala S., Bhatia M. (2005). Chemokines in acute respiratory distress syndrome. Am. J. Physiol. Lung Cell Mol. Physiol..

[12] Coperchini F., Chiovato L., Croce L., Magri F., Rotondi M. (2020). The cytokine storm in COVID-19: An overview of the involvement of the chemokine/chemokine-receptor system. Cytokine Growth Factor Rev..

[13] Hoffmann M., Kleine-Weber H., Schroeder S., Krüger N., Herrler T., Erichsen S., Schiergens T.S., Herrler G., Wu N.H., Nitsche A. (2020). SARS-CoV-2 Cell Entry Depends on ACE2 and TMPRSS2 and Is Blocked by a Clinically Proven Protease Inhibitor. Cell.

[14] Clausen T.M., Sandoval D.R., Spliid C.B., Pihl J., Perrett H.R., Painter C.D., Narayanan A., Majowicz S.A., Kwong E.M., McVicar R.N. (2020). SARS-CoV-2 Infection Depends on Cellular Heparan Sulfate and ACE2. Cell.

[15] Hu B., Huang S., Yin L. (2020). The cytokine storm and COVID-19. J. Med. Virol..

[16] Hirano T., Murakami M. (2020). COVID-19: A New Virus, but a Familiar Receptor and Cytokine Release Syndrome. Immunity.

[17] Murakami M., Kamimura D., Hirano T. (2019). Pleiotropy and Specificity: Insights from the Interleukin 6 Family of Cytokines. Immunity.

[18] Han S., Mallampalli R.K. (2015). The acute respiratory distress syndrome: From mechanism to translation. J. Immunol..

[19] Ranieri V.M., Rubenfeld G.D., Thompson B.T., Ferguson N.D., Caldwell E., Fan E., Camporota L., Slutsky A.S. (2012). Acute respiratory distress syndrome: The Berlin Definition. JAMA.

[20] Meduri G.U., Annane D., Chrousos G.P., Marik P.E., Sinclair S.E. (2009). Activation and regulation of systemic inflammation in ARDS: Rationale for prolonged glucocorticoid therapy. Chest.

[21] Matthay M.A., Zimmerman G.A. (2005). Acute Lung Injury and the Acute Respiratory Distress Syndrome: Four Decades of Inquiry into Pathogenesis and Rational Management. Am. J. Respir. Cell Mol. Biol..

[22] Matthay M.A., Zemans R.L. (2011). The Acute Respiratory Distress Syndrome: Pathogenesis and Treatment. Annu. Rev. Pathol..

[23] Zimmerman N.P., Vongsa R.A., Wendt M.K., Dwinell M.B. (2008). Chemokines and chemokine receptors in mucosal homeostasis at the intestinal epithelial barrier in inflammatory bowel disease. Inflamm. Bowel Dis..

[24] Kawashima D., Oshitani N., Jinno Y., Watanabe K., Nakamura S., Higuchi K., Arakawa T. (2005). Augmented expression of secondary lymphoid tissue chemokine and EBI1 ligand chemokine in Crohn’s disease. J. Clin. Pathol..

[25] Papadakis K.A., Prehn J., Moreno S.T., Cheng L., Kouroumalis E.A., Deem R., Breaverman T., Ponath P.D., Andrew D.P., Green P.H. (2001). CCR9-positive lymphocytes and thymus-expressed chemokine distinguish small bowel from colonic Crohn’s disease. Gastroenterology.

[26] Ogawa H., Iimura M., Eckmann L., Kagnoff M.F. (2004). Regulated production of the chemokine CCL28 in human colon epithelium. Am. J. Physiol. Gastrointest. Liver Physiol..

[27] Sørensen T.L., Tani M., Jensen J., Pierce V., Lucchinetti C., Folcik V.A., Qin S., Rottman J., Sellebjerg F., Strieter R.M. (1999). Expression of specific chemokines and chemokine receptors in the central nervous system of multiple sclerosis patients. J. Clin. Investig..

[28] Barlic J., Murphy P.M. (2007). Chemokine regulation of atherosclerosis. J. Leukoc. Biol..

[29] Nelken N.A., Coughlin S.R., Gordon D., Wilcox J.N. (1991). Monocyte chemoattractant protein-1 in human atheromatous plaques. J. Clin. Investig..

[30] Xiong Y., Liu Y., Cao L., Wang D., Guo M., Guo D., Hu W., Yang J., Tang Z., Zhang Q. (2020). Transcriptomic characteristics of bronchoalveolar lavage fluid and peripheral blood mononuclear cells in COVID-19 patients. Emerg. Microbes Infect..

[31] Zhao Y., Qin L., Zhang P., Li K., Liang L., Sun J., Xu B., Dai Y., Li X., Zhang C. (2020). Longitudinal COVID-19 profiling associates IL-1RA and IL-10 with disease severity and RANTES with mild disease. JCI Insight.

[32] Li S., Jiang L., Li X., Lin F., Wang Y., Li B., Jiang T., An W., Liu S., Liu H. (2020). Clinical and pathological investigation of patients with severe COVID-19. JCI Insight.

[33] Yan Y., Yang Y., Wang F., Ren H., Zhang S., Shi X., Yu X., Dong K. (2020). Clinical characteristics and outcomes of patients with severe covid-19 with diabetes. BMJ Open Diabetes Res. Care.

[34] McElvaney O.J., McEvoy N.L., McElvaney O.F., Carroll T.P., Murphy M.P., Dunlea D.M., Ní Choileáin O., Clarke J., O’Connor E., Hogan G. (2020). Characterization of the Inflammatory Response to Severe COVID-19 Illness. Am. J. Respir. Crit. Care Med..

[35] Del Valle D.M., Kim-Schulze S., Huang H.-H., Beckmann N.D., Nirenberg S., Wang B., Lavin Y., Swartz T.H., Madduri D., Stock A. (2020). An inflammatory cytokine signature predicts COVID-19 severity and survival. Nat. Med..

[36] Zhang X., Tan Y., Ling Y., Lu G., Liu F., Yi Z., Jia X., Wu M., Shi B., Xu S. (2020). Viral and host factors related to the clinical outcome of COVID-19. Nature.

[37] Xu X., Yu M.-Q., Shen Q., Wang L.-Z., Yan R.-D., Zhang M.-Y., Liu J.-Y., Qu Y.-Q. (2020). Analysis of inflammatory parameters and disease severity for 88 hospitalized COVID-19 patients in Wuhan, China. Int. J. Med. Sci..

[38] Nagant C., Ponthieux F., Smet J., Dauby N., Doyen V., Besse-Hammer T., De Bels D., Maillart E., Corazza F. (2020). A score combining early detection of cytokines accurately predicts COVID-19 severity and intensive care unit transfer. Int. J. Infect. Dis..

[39] Laing A.G., Lorenc A., Del Molino Del Barrio I., Abhishek D., Matthew F., Leticia M., Muñoz-Ruiz M., McKenzie D.R., Hayday T.S., Francos-Quijorna I. (2020). A dynamic COVID-19 immune signature includes associations with poor prognosis. Nat. Med..

[40] Chi Y., Ge Y., Wu B., Zhang W., Wu T., Wen T., Liu J., Guo X., Huang C., Jiao Y. (2020). Serum Cytokine and Chemokine Profile in Relation to the Severity of Coronavirus Disease 2019 in China. J. Infect. Dis..

[41] Petrey A.C., Qeadan F., Middleton E.A., Pinchuk I.V., Campbell R.A., Beswick E.J. (2020). Cytokine release syndrome in COVID-19: Innate immune, vascular, and platelet pathogenic factors differ in severity of disease and sex. J. Leukoc. Biol..

[42] Sims J.T., Krishnan V., Chang C.-Y., Engle S.M., Casalini G., Rodgers G.H., Bivi N., Nickoloff B.J., Konrad R.J., De Bono S. (2020). Characterization of the cytokine storm reflects hyperinflammatory endothelial dysfunction in COVID-19. J. Allergy Clin. Immunol..

[43] Chen Y., Wang J., Liu C., Su L., Zhang D., Fan J., Yang Y., Xiao M., Xie J., Xu Y. (2020). IP-10 and MCP-1 as biomarkers associated with disease severity of COVID-19. Mol. Med..

[44] Yang Y., Shen C., Li J., Yuan J., Wei J., Huang F., Wang F., Li G., Li Y., Xing L. (2020). Plasma IP-10 and MCP-3 levels are highly associated with disease severity and predict the progression of COVID-19. J. Allergy Clin. Immunol..

[45] Patterson B.K., Seethamraju H., Dhody K., Corley M.J., Kazempour K., Lalezari J.P., Pang A.P., Sugai C., Francisco E.B., Pise A. (2020). Disruption of the CCL5/RANTES-CCR5 Pathway Restores Immune Homeostasis and Reduces Plasma Viral Load in Critical COVID-19. medRxiv.

[46] Mahler D.A., Huang S., Tabrizi M., Bell G.M. (2004). Efficacy and safety of a monoclonal antibody recognizing interleukin-8 in COPD: A pilot study. Chest.

[47] Rennard S.I., Fogarty C., Kelsen S., Long W., Ramsdell J., Allison J., Mahler D., Saadeh C., Siler T., Snell P. (2007). The safety and efficacy of infliximab in moderate to severe chronic obstructive pulmonary disease. Am. J. Respir. Crit. Care Med..

[48] Hehlgans T., Pfeffer K. (2005). The intriguing biology of the tumour necrosis factor/tumour necrosis factor receptor superfamily: Players, rules and the games. Immunology.

[49] Lefebvre A.L., McAuliffe L. (2016). Targeted Immunomodulatory Therapy: An Overview. Rhode Isl. Med. J..

[50] Maini R.N., Elliott M.J., Brennan F.M., Feldmann M. (1995). Beneficial effects of tumour necrosis factor-alpha (TNF-alpha) blockade in rheumatoid arthritis (RA). Clin. Exp. Immunol..

[51] Monaco C., Nanchahal J., Taylor P., Feldmann M. (2015). Anti-TNF therapy: Past, present and future. Int. Immunol..

[52] Brenner E.J., Ungaro R.C., Colombel J.F., Kappelman M.D. SECURE-IBD Database Public Data Update. https://covidibd.org/.

[53] Abdullah A., Neurath M.F., Atreya R. (2020). Mild COVID-19 Symptoms in an Infliximab-Treated Ulcerative Colitis Patient: Can Ongoing Anti-TNF Therapy Protect against the Viral Hyperinflammatory Response and Avoid Aggravated Outcomes?. Visc. Med..

[54] Stallmach A., Kortgen A., Gonnert F., Coldewey S.M., Reuken P., Bauer M. (2020). Infliximab against severe COVID-19-induced cytokine storm syndrome with organ failure-a cautionary case series. Crit. Care.

[55] Richards D. (2020). Adalimumab for Coronavirus in Community Care. http://isrctn.com.

[56] Cavalli G., de Luca G., Campochiaro C., Della-Torre E., Ripa M., Canetti D., Oltolini C., Castiglioni B., Din C.T., Boffini N. (2020). Interleukin-1 blockade with high-dose anakinra in patients with COVID-19, acute respiratory distress syndrome, and hyperinflammation: A retrospective cohort study. Lancet Rheumatol..

[57] Huet T., Beaussier H., Voisin O., Jouveshomme S., Dauriat G., Lazareth I., Sacco E., Naccache J.-M., Bézie Y., Laplanche S. (2020). Anakinra for severe forms of COVID-19: A cohort study. Lancet Rheumatol..

[58] Langer-Gould A., Smith J.B., Gonzales E.G., Castillo R.D., Figueroa J.G., Ramanathan A., Li B.H., Gould M.K. (2020). Early identification of COVID-19 cytokine storm and treatment with anakinra or tocilizumab. Int. J. Infect. Dis..

[59] Navarro-Millán I., Sattui S.E., Lakhanpal A., Zisa D., Siegel C.H., Crow M.K. (2020). Use of Anakinra to Prevent Mechanical Ventilation in Severe COVID-19: A Case Series. Arthritis Rheumatol..

[60] Nemchand P., Tahir H., Mediwake R., Lee J. (2020). Cytokine storm and use of anakinra in a patient with COVID-19. BMJ Case Rep..

[61] Aouba A., Baldolli A., Geffray L., Verdon R., Bergot E., Martin-Silva N., Justet A. (2020). Targeting the inflammatory cascade with anakinra in moderate to severe COVID-19 pneumonia: Case series. Ann. Rheum. Dis..

[62] Cauchois R., Koubi M., Delarbre D., Manet C., Carvelli J., Blasco V.B., Jean R., Fouche L., Bornet C., Pauly V. (2020). Early IL-1 receptor blockade in severe inflammatory respiratory failure complicating COVID-19. Proc. Natl. Acad. Sci. USA.

[63] Ucciferri C., Auricchio A., Di Nicola M., Potere N., Abbate A., Cipollone F., Vecchiet J., Falasca K. (2020). Canakinumab in a subgroup of patients with COVID-19. Lancet Rheumatol..

[64] Brocker C., Thompson D., Matsumoto A., Nebert D.W., Vasiliou V. (2010). Evolutionary divergence and functions of the human interleukin (IL) gene family. Hum. Genom..

[65] Copaescu A., Smibert O., Gibson A., Phillips E.J., Trubiano J.A. (2020). The role of IL-6 and other mediators in the cytokine storm associated with SARS-CoV-2 infection. J. Allergy Clin. Immunol..

[66] Zhang C., Wu Z., Li J.-W., Zhao H., Wang G.-Q. (2020). Cytokine release syndrome in severe COVID-19: Interleukin-6 receptor antagonist tocilizumab may be the key to reduce mortality. Int. J. Antimicrob. Agents.

[67] Han H., Ma Q., Li C., Liu R., Zhao L., Wang W., Zhang P., Liu X., Gao G., Liu F. (2020). Profiling serum cytokines in COVID-19 patients reveals IL-6 and IL-10 are disease severity predictors. Emerg. Microbes Infect..

[68] Kotch C., Barrett D., Teachey D.T. (2019). Tocilizumab for the treatment of chimeric antigen receptor T cell-induced cytokine release syndrome. Expert Rev. Clin. Immunol..

[69] Yokota S., Imagawa T., Mori M., Miyamae T., Aihara Y., Takei S., Iwata N., Umebayashi H., Murata T., Miyoshi M. (2008). Efficacy and safety of tocilizumab in patients with systemic-onset juvenile idiopathic arthritis: A randomized, double-blind, placebo-controlled, withdrawal phase III trial. Lancet.

[70] Kaly L., Rosner I. (2012). Tocilizumab—A novel therapy for non-organ-specific autoimmune diseases. Best Pract. Res. Clin. Rheumatol..

[71] Lan S.-H., Lai C.-C., Huang H.-T., Chang S.-P., Lu L.-C., Hsueh P.-R. (2020). Tocilizumab for severe COVID-19: A systematic review and meta-analysis. Int. J. Antimicrob. Agents.

[72] Boregowda U., Perisetti A., Nanjappa A., Gajendran M., Kutti Sridharan G., Goyal H. (2020). Addition of Tocilizumab to the Standard of Care Reduces Mortality in Severe COVID-19: A Systematic Review and Meta-Analysis. Front. Med..

[73] Capra R., Rossi N., de Mattioli F., Romanelli G., Scarpazza C., Sormani M.P., Cossi S. (2020). Impact of low dose tocilizumab on mortality rate in patients with COVID-19 related pneumonia. Eur. J. Intern. Med..

[74] Klopfenstein T., Zayet S., Lohse A., Balblanc J.-C., Badie J., Royer P.-Y., Toko L., Mezher C., Kadiane-Oussou N.J., Bossert M. (2020). Tocilizumab therapy reduced intensive care unit admissions and/or mortality in COVID-19 patients. Med. Mal. Infect..

[75] Ramaswamy M., Mannam P., Comer R., Sinclair E., McQuaid D.B., Schmidt M.L. (2020). Off-Label Real World Experience Using Tocilizumab for Patients Hospitalized with COVID-19 Disease in a Regional Community Health System: A Case-Control Study. medRxiv.

[76] Roumier M., Paule R., Groh M., Vallee A., Ackermann F. (2020). Interleukin-6 blockade for severe COVID-19. medRxiv.

[77] Wadud N., Ahmed N., Shergil M.M., Khan M., Krishna M.G., Gilani A., el Zarif S., Galaydick J., Linga K., Koor S. (2020). Improved survival outcome in SARs-CoV-2 (COVID-19) Acute Respiratory Distress Syndrome patients with Tocilizumab administration. medRxiv.

[78] Morena V., Milazzo L., Oreni L., Bestetti G., Fossali T., Bassoli C., Torre A., Cossu M.V., Minari C., Ballone E. (2020). Off-label use of tocilizumab for the treatment of SARS-CoV-2 pneumonia in Milan, Italy. Eur. J. Intern. Med..

[79] Toniati P., Piva S., Cattalini M., Garrafa E., Regola F., Castelli F., Franceschini F., Airò P., Bazzani C., Beindorf E.-A. (2020). Tocilizumab for the treatment of severe COVID-19 pneumonia with hyperinflammatory syndrome and acute respiratory failure: A single center study of 100 patients in Brescia, Italy. Autoimmun. Rev..

[80] Alattar R., Ibrahim T.B.H., Shaar S.H., Abdalla S., Shukri K., Daghfal J.N., Khatib M.Y., Aboukamar M., Abukhattab M., Alsoub H.A. (2020). Tocilizumab for the treatment of severe coronavirus disease 2019. J. Med. Virol..

[81] Sciascia S., Aprà F., Baffa A., Baldovino S., Boaro D., Boero R., Bonora S., Calcagno A., Cecchi I., Cinnirella G. (2020). Pilot prospective open, single-arm multicentre study on off-label use of tocilizumab in patients with severe COVID-19. Clin. Exp. Rheumatol..

[82] Guillén L., Padilla S., Fernández M., Agulló V., García J.A., Telenti G., García-Abellán J., Botella Á., Gutiérrez F., Masiá M. (2020). Preemptive interleukin-6 blockade in patients with COVID-19. Sci. Rep..

[83] Kewan T., Covut F., Al-Jaghbeer M.J., Rose L., Gopalakrishna K.V., Akbik B. (2020). Tocilizumab for treatment of patients with severe COVID-19: A retrospective cohort study. EClinicalMedicine.

[84] Price C.C., Altice F.L., Shyr Y., Alan K., Lauren P., George G., Marwan M.A., Dayna M., Sheau-Chiann C., Shana E.G. (2020). Tocilizumab Treatment for Cytokine Release Syndrome in Hospitalized Patients With Coronavirus Disease 2019: Survival and Clinical Outcomes. Chest.

[85] Stone J.H., Frigault M.J., Serling-Boyd N.J., Fernandes A.D., Harvey L., Foulkes A.S., Horick N.K., Healy B.C., Shah R., Bensaci A.M. (2020). Efficacy of Tocilizumab in Patients Hospitalized with Covid-19. N. Engl. J. Med..

[86] Raimondo M.G., Biggioggero M., Crotti C., Becciolini A., Favalli E.G. (2017). Profile of sarilumab and its potential in the treatment of rheumatoid arthritis. Drug Des. Dev. Ther..

[87] Gremese E., Cingolani A., Bosello S.L., Alivernini S., Tolusso B., Perniola S., Landi F., Pompili M., Murri R., Santoliquido A. (2020). Sarilumab use in severe SARS-CoV-2 pneumonia. EClinicalMedicine.

[88] Benucci M., Giannasi G., Cecchini P., Gobbi F.L., Damiani A., Grossi V., Infantino M., Manfredi M. (2020). COVID-19 pneumonia treated with Sarilumab: A clinical series of eight patients. J. Med. Virol..

[89] Della-Torre E., Campochiaro C., Cavalli G., De Luca G., Napolitano A., La Marca S., Boffini N., Da Prat V., Di Terlizzi G., Lanzillotta M. (2020). Interleukin-6 blockade with sarilumab in severe COVID-19 pneumonia with systemic hyperinflammation: An open-label cohort study. Ann. Rheum. Dis..

[90] Montesarchio V., Parrella R., Iommelli C., Bianco A., Manzillo E., Fraganza F., Palumbo C., Rea G., Murino P., De Rosa R. (2020). Outcomes and biomarker analyses among patients with COVID-19 treated with interleukin 6 (IL-6) receptor antagonist sarilumab at a single institution in Italy. J. Immunother. Cancer.

[91] Sarosiek S., Shah R., Munshi N.C. (2016). Review of siltuximab in the treatment of multicentric Castleman’s disease. Ther. Adv. Hematol..

[92] Gritti G., Raimondi F., Ripamonti D., Riva I., Landi F., Alborghetti L., Frigeni M., Damiani M., Micò C., Fagiuoli S. (2020). Use of siltuximab in patients with COVID-19 pneumonia requiring ventilatory support. medRxiv.

[93] Cheloff A.Z., Al-Samkari H. (2020). Emapalumab for the treatment of hemophagocytic lymphohistiocytosis. Drugs Today.

[94] Xu Z., Shi L., Wang Y., Zhang J., Huang L., Zhang C., Liu S., Zhao P., Liu H., Zhu L. (2020). Pathological findings of COVID-19 associated with acute respiratory distress syndrome. Lancet Respir. Med..

[95] Stebbing J., Phelan A., Griffin I., Tucker C., Oechsle O., Smith D., Richardson P. (2020). COVID-19: Combining antiviral and anti-inflammatory treatments. Lancet Infect. Dis..

[96] Stebbing J., Sánchez Nievas G., Falcone M., Youhanna S., Richardson P., Ottaviani S., Shen J.X., Sommerauer C., Tiseo G., Ghiadoni L. (2020). JAK inhibition reduces SARS-CoV-2 liver infectivity and modulates inflammatory responses to reduce morbidity and mortality. Sci. Adv..

[97] Gozzetti A., Capochiani E., Bocchia M. (2020). The Janus kinase 1/2 inhibitor ruxolitinib in COVID-19. Leukemia.

[98] La Rosée F., Bremer H.C., Gehrke I., Kehr A., Hochhaus A., Birndt S., Fellhauer M., Henkes M., Kumle B., Russo S.G. (2020). The Janus kinase 1/2 inhibitor ruxolitinib in COVID-19 with severe systemic hyperinflammation. Leukemia.

[99] Yeleswaram S., Smith P., Burn T., Covington M., Juvekar A., Li Y., Squier P., Langmuir P. (2020). Inhibition of cytokine signaling by ruxolitinib and implications for COVID-19 treatment. Clin. Immunol..

[100] Barmania F., Pepper M.S. (2013). C-C chemokine receptor type five (CCR5): An emerging target for the control of HIV infection. Appl. Transl. Genom..

[101] Dragic T., Litwin V., Allaway G.P., Martin S.R., Huang Y., Nagashima K.A., Cayanan C.S., Maddon P.J., Koup R.A., Moore J.P. (1996). HIV-1 entry into CD4+ cells is mediated by the chemokine receptor CC-CKR-5. Nature.

[102] Ray N. (2009). Maraviroc in the treatment of HIV infection. Drug Des. Dev. Ther..

[103] Xu Z.-S., Shu T., Kang L., Wu D., Zhou X., Liao B.-W., Sun X.-L., Zhou X., Wang Y.-Y. (2020). Temporal profiling of plasma cytokines, chemokines and growth factors from mild, severe and fatal COVID-19 patients. Signal. Transduct. Target. Ther..

[104] Law H.K.W., Cheung C.Y., Ng H.Y., Sia S.F., Chan Y.O., Luk W., Nicholls J.M., Peiris J.S.M., Lau Y.L. (2005). Chemokine up-regulation in SARS-coronavirus–infected, monocyte-derived human dendritic cells. Blood.

[105] Yen Y.-T., Liao F., Hsiao C.-H., Kao C.-L., Chen Y.-C., Wu-Hsieh B.A. (2006). Modeling the early events of severe acute respiratory syndrome coronavirus infection in vitro. J. Virol..

[106] Akalin E., Azzi Y., Bartash R., Seethamraju H., Parides M., Hemmige V., Ross M., Forest S., Goldstein Y.D., Ajaimy M. (2020). Covid-19 and Kidney Transplantation. N. Engl. J. Med..

[107] Yang B., Fulcher J.A., Ahn J., Berro M., Goodman-Meza D., Dhody K., Sacha J.B., Naeim A., Yang O.O. (2020). Clinical Characteristics and Outcomes of COVID-19 Patients Receiving Compassionate Use Leronlimab. Clin. Infect. Dis..

[108] Henry B., Cheruiyot I., Vikse J., Mutua V., Kipkorir V., Benoit J., Plebani M., Bragazzi N., Lippi G. (2020). Lymphopenia and neutrophilia at admission predicts severity and mortality in patients with COVID-19: A meta-analysis. Acta Biomed..

[109] Somers E.C., Eschenauer G.A., Troost J.P., Golob J.L., Gandhi T.N., Wang L., Zhou N., Petty L.A., Baang J.H., Dillman N.O. (2020). Tocilizumab for treatment of mechanically ventilated patients with COVID-19. medRxiv.

[110] Scherger S., Henao-Martínez A., Franco-Paredes C., Shapiro L. (2020). Rethinking interleukin-6 blockade for treatment of COVID-19. Med. Hypotheses.

[111] Kimmig L.M., Wu D., Gold M., Pettit N.N., Pitrak D., Mueller J., Husain A.N., Mutlu E.A., Mutlu G.M. (2020). IL-6 Inhibition in Critically Ill COVID-19 Patients Is Associated With Increased Secondary Infections. Front. Med..

